# Mussel processing wastewater: a low-cost substrate for the production of astaxanthin by *Xanthophyllomyces dendrorhous*

**DOI:** 10.1186/s12934-015-0375-5

**Published:** 2015-11-09

**Authors:** Isabel Rodríguez Amado, José Antonio Vázquez

**Affiliations:** Grupo de Reciclado y Valorización de Residuos (REVAL), Instituto de Investigacións Mariñas (IIM-CSIC), R/Eduardo Cabello 6, 36208 Vigo, Spain; Departamento de Química Analítica y Alimentaria, Facultad de Ciencias de Ourense, Universidad de Vigo, Campus As Lagoas s/n, Orense, Spain

**Keywords:** Astaxanthin, Yeast, Antioxidant, Glycogen, Fermentation, Aquaculture, Amylolytic activity, Marine by-products

## Abstract

**Background:**

The use of astaxanthin in different industries such as the chemical, pharmaceutical, food, animal feed and cosmetic has been receiving increasing attention in recent years. Natural supplies of the pigment include crustacean by-products, algal, and microbial cultivation, being the yeast *Xanthophyllomyces dendrorhous* together with the alga *Haematococcus pluvialis* the most promising microorganisms for this bioproduction. Different vegetable by-products of the food industry have been explored so far as low-cost substrates for the production of astaxanthin by *X. dendrorhous.* This study focuses for the first time on the use of a low-cost formulated medium from a marine by-product, mussel-processing wastewater, for the production of astaxanthin by the yeast *X. dendrorhous*.

**Results:**

The yeast was able to grow in non-saccharified mussel broth, revealing the ability of the microorganism to hydrolyze glycogen. However, partial glycogen saccharification with α-amylase was needed for astaxanthin biosynthesis, obtaining maximal productions of 22.5–26.0 mg/L towards the end of the culture and coinciding with yeast highest amylolytic activity. Cultivations in totally-saccharified media revealed an increase in maximal cell concentrations and a decrease in maximal growth rates and astaxanthin production with increasing glucose initial concentration.

**Conclusions:**

Astaxanthin production was higher in partially-saccharified mussel-processing waste than in synthetic medium (yeast peptone dextrose) containing glucose as carbon source (13 mg/L), suggesting this by-product is a promising nutritive medium for astaxanthin production. The use of this effluent also contributes towards the recycling and depuration of this highly pollutant effluent.

## Background

Astaxanthin (3,3-dihydroxy-β,β-carotene-4,4 dione) is a ketocarotene widely used in aquaculture as a feed additive for the pigmentation of salmonid meat, and crustacean shells [1]. Astaxanthin has been reported to inhibit lipid peroxidation and low-density lipoprotein (LDL) oxidation [2] in rats and mice due to its potent antioxidant activity. Recent studies suggest astaxanthin could improve the serum lipid profile [3], and enhance the cytotoxic activity of natural killer cells [4]. As a consequence of these potential benefits for human health, the market for astaxanthin as a dietary supplement in human diets has increased in the last years [5].

Several natural sources of astaxanthin have been explored so far owing to its increasing level of demand for novel applications in the food, pharmaceutical and cosmetic industries [6]. Natural supplies of the pigment include shrimp [7], crawfish by-products [8], crustacean wastewaters [9, 10] and microbial cultivation [11]. The synthesis of astaxanthin by several yeast species belonging to the genera *Rhodotorula* and *Phaffia*, and by the microalgae *Chlorella zofingiensis*, *Chlorococcum* sp. and *Haematococcus pluvialis* has led to consider these microorganisms as potential pigment sources [5]. Among them, *Xanthophyllomyces dendrorhous* (former *Phaffia rhodozyma*), and *H. pluvialis* are to date the most promising microorganisms, being the alga the largest producer of the pigment. In fact, astaxanthin for aquaculture and human dietary supplements are produced from *H. pluvialis* at industrial scale [12].

Although astaxanthin production from yeasts is lower than algae [13], the former is preferred due to higher growth rates and easier cultivation conditions [14] that might decrease the production time at industrial scale [1]. Different agri-food wastes such as cassava starch [15], corn fiber [16], molasses [17], and eucalyptus globules wood [18] were explored as astaxanthin low-cost sources. However to the best of our knowledge, marine by-products have not yet been investigated for the cultivation of *X. dendrorhous* in spite of their high content of nutritive compounds. Mussel processing wastewater (MPW) is a residual effluent from the canning industry rich in glycogen and proteins [19]. This by-product was successfully utilized to produce amylase [20], bacteriocins [21], and hyaluronic acid [22] by different microorganisms.

The ability to degrade starch is not widespread in yeast, however, *X. dendrorhous* produces a β-amylase whose synthesis is induced by starch and maltose [23]. Also shows extracellular exo-acting enzymatic activity able to cleave α 1-4 glycosidic bonds from soluble starch, maltose, and maltooligosaccharides [24]. Despite its unusual substrate specificity, the enzyme was classified as α-glucosidase due the formation of glucose, and not maltose or maltotriose, as the final product of the hydrolytic reaction. Those enzymes, with potential interesting industrial applications [23, 25], would enable the use of amylaceous substrates as carbon sources for astaxanthin production by *X. dendrorhous*.

In the present study, we assessed the suitability of concentrated and partially or totally-saccharified mussel processing wastewater for the production of astaxanthin in submerged culture of *X. dendrorhous*.

## Results and discussion

### Selection of astaxanthin-producing strain

**Fig. 1:**
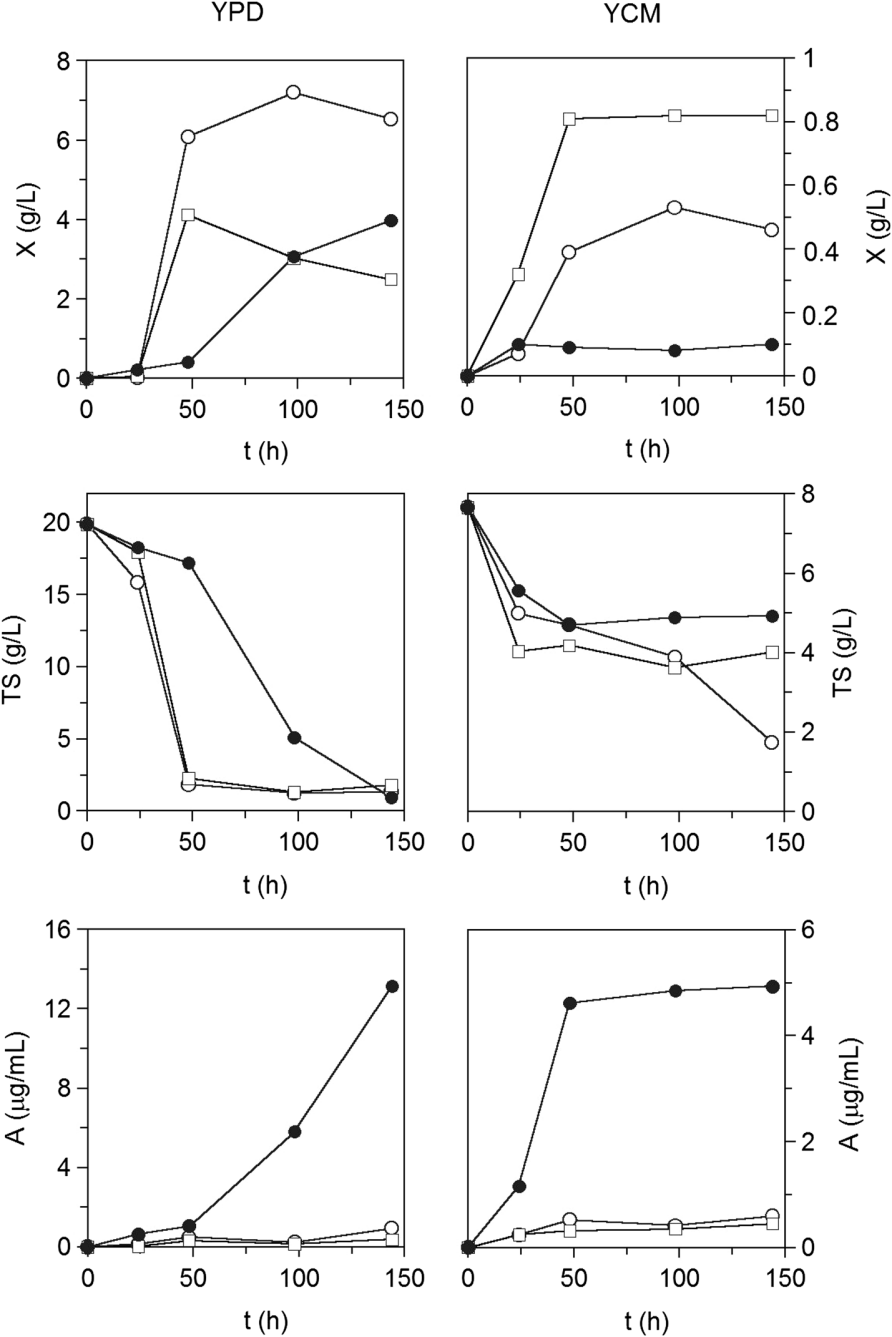
Cultivation of *X. dendrorhous* in YPD and YMC media. *Open circle* CECT 1690, *open square* CECT 11028, *filled circle* ATCC 74219. *X* biomass, *TS* total sugars, *A* astaxanthin

Astaxanthin-producing strains CECT 1690, CECT 11028 and ATCC 74219 were screened in Yeast Peptone Dextrose (YPD) and Yeast Complex Medium (YCM, Table 1). All three strains were able to metabolize both glucose and starch, confirming *X. dendrorhous* amylolytic activity. Growth and astaxanthin production in YCM were lower due to incomplete sugar consumption (Fig. 1). The microorganism only consumed around 50 % starch of the culture medium after 4 days of culture. *X. dendrorhous* showed a similar trend of starch consumption when grown in MM (0.7 % yeast nitrogen base) medium supplemented with 1 % starch as a carbon source [24]. These authors found glucose was the only product from hydrolytic reactions, pointing to yeast α-glucosidase activity. Díaz et al. [23] also found yeast amylolytic activity in SD (0.7 % yeast nitrogen base without amino acids) medium containing 1 % starch. However, they found the enzyme was a β-amylase, due to the absence of glucose as the final product of the hydrolytic activity.

**Table 1:** Composition of the culture media used in the present study (g/L)

	YPD	YCM	MS	PSM	PSMS	TSMS
Glucose	20.00	–	–	50	50	20/40/80/100
Total sugars	–	–	100	100	100	20/40/80/100
Soluble starch	–	5.00	–	–	–	–
Bactopeptone	20.00	–	–	–	–	–
Yeast extract	10.00	2.50	10.00	10.00	10.00	10.00
Malt extract	–	2.50	–	–	–	–
(NH_4_)_2_SO_4_	–	–	5.00	5.00	5.00	5.00
KH_2_PO_4_	–	1.00	2.00	2.00	2.00	2.00
MgSO_4_	–	0.50	1.00	–	1.00	1.00
K_2_HPO_4_	–	–	0.40	0.40	0.40	0.40
CaCl_2_	–	0.35	0.20	–	0.20	0.20
ZnSO_4_	–	–	0.03	–	0.03	0.03
FeNH_4_(SO_4_)_2_	–	–	0.01	–	0.01	0.01
CuSO_4_	–	–	0.002	–	0.002	0.002
NH_4_Cl	–	2.00	–	–	–	–
Vitamin premix (% v/v)	–	–	5	–	5	5
C/N ratio	6.33	10.6	35.7	35.3	35.5	12.0/20.5/33.7/38.1

The vitamin premix contains 2 g/L inositol, 1 g/L pyridoxine, 0.2 g/L calcium pantothenate, 40 mg/L thiamine and 2 mg/L biotin

*YPD* yeast peptone dextrose, *YCM* yeast complet medium, *MS* mussel supplemented medium, *PSM* partially saccharified mussel medium, *PSMS* partially saccharified mussel supplemented medium, *TSMS* totally saccharified mussel supplemented medium

Astaxanthin productions were higher for strain ATCC 74219 irrespective of the carbon source. ATCC 74219 productions were fivefold to more than tenfold higher in starch and glucose containing media, respectively compared to strains CECT 1690 and CECT 11028 (Fig. 1). Astaxanthin biosynthesis was mainly associated with the second half of the culture cycle (exponential and stationary phase), reaching maximum productions (13 mg/L) at the end of the culture (Fig. 1). Carotenoid production by *X. dendrorhous* typically shows a pattern of a secondary metabolite [26]. While astaxanthin production in ATCC 74219 was the highest among the three *X. dendrorhous* strains, biomass production was the lowest. These results agree with the greater yields of astaxanthin on biomass (*Y*_*A/X*_) compared to CECT 1690 and CECT 11028 strains (Table 2). Uncoupling of biomass and astaxanthin production is usually attributed to different cell nutritional requirements, where a low C/N ratio improves cell biosynthesis while a high C/N ratio enhances astaxanthin production [27, 28]. Astaxanthin yields were, in fact, higher in the culture medium with the highest C/N ratio (Table 1).

**Table 2:** Yields of *X. dendrorhous* cultivation in YPD and YMC culture media

	*Y*_*A/X*_ (mg/g)	*Y*_*A/TS*_ (mg/g)	*Y*_*X/TS*_ (g/g)
*YPD*			
CECT1690	0.143	0.050	0.352
CECT11028	0.148	0.020	0.138
ATCC 74219	3.312	0.693	0.209
*YCM*			
CECT1690	1.263	0.099	0.078
CECT11028	0.554	0.124	0.224
ATCC 74219	48.70	1.806	0.037

*Y*_*A/X*_ yield of astaxanthin production on biomass, *Y*_*A/TS*_ yield of astaxanthin production on sugars consumed, *Y*_*X/TS*_ yield of biomass on sugars consumed

According to these results, the strain ATCC 74219 was selected for further investigation due to its higher astaxanthin production and to its ability to grow in starchy substrates.

### Selection of astaxanthin extraction method

**Fig. 2:**
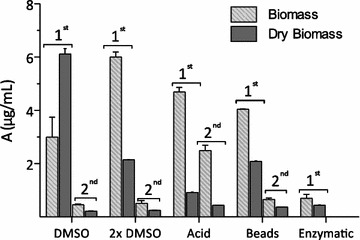
Astaxanthin recovery from *X. dendrorhous* ATCC 74219 using different cell disruption methods applied to fresh and dried yeast cells. First and second extractions were carried out in hexane:ethylacetate (1:1). Mean values and standard deviations from duplicate samples are shown

The most effective method of carotenoid extraction was cell disruption with dimethylsulfoxide (DMSO) followed by autoclaving in acidic conditions, mechanical abrasion and enzymatic treatment (Fig. 2). Disruption of yeast cells using DMSO improved astaxanthin recovery according to previous papers [29].

In general, astaxanthin was more effectively released from fresh cells, and a second extraction with hexane: ethylacetate did not improve astaxanthin recovery (Fig. 2). Cell disruption was more effective on dried cells, as observed by Da Fonseca et al. [29] using a similar biomass/DMSO ratio. Astaxanthin recovery was maximal (6 mg/L) from fresh cells and high biomass/DMSO ratio, and dried biomass using low biomass/DMSO (Fig. 2). When using high-density cell (>2 × 10^8^ cells) cultures [30], such as those of the present work (3 × 10^8^ cells), the extraction may be incomplete. This lower recovery is because the volume of the chemical disruptor is too low, leading to the formation of a thick insoluble pigmented interface keeping the carotenoids [30]. The maximum astaxanthin specific concentration obtained in this study (2 mg/g) was higher compared to the 332–786 µg/g obtained by Sedmak et al. [30] from various strains of *X. dendrorhous* using the same DMSO extraction method. Da Fonseca et al. [29] reported slightly higher astaxanthin recoveries (2.5 mg/g) from dried and freeze-dried yeast cells after several extraction cycles (2–4) with DMSO.

According to these results, the method of astaxanthin extraction selected for further pigment recovery was DMSO (×2) applied to fresh yeast cells, avoiding the need for drying pretreatment.

### Culture of *Xanthophyllomyces dendrorhous* in partially-saccharified mussel wastewater

Three nutritive broths were formulated using autoclaved mussel-processing waste as culture media for astaxanthin production by ATCC 74219. The use of high molecular weight polysaccharides as carbon source requires the synthesis of yeast extracellular amylases, which could be a restrictive factor for the utilization of an effluent containing glycogen as the carbon source [31]. A partial saccharification using α-amylase from *Aspergillus oryzae* was carried out under previously optimized conditions [31], leading to a 51 % of hydrolysis referred to the level of reducing sugars. Salts and vitamins reported to improve carotenoid production were added to culture media containing non-saccharified (MS) or partially-saccharified (PSMS) mussel processing waste. A bibliographic review revealed formulations containing inorganic nitrogen and phosphorus sources ((NH_4_)_2_SO_4_, K_2_HPO_4_, and KH_2_PO_4_) and mesonutrients such as MgSO_4_ and CaCl_2_ lead to high astaxanthin productions (>5000 µg/g) in semi-industrial [26], and industrial [27] submerged cultures. According to the latter, including micronutrients (FeNH_4_(SO_4_)_2_, CuSO_4_ and ZnSO_4_) and traces of vitamins (inositol, pyridoxine, calcium pantothenate, thiamine, and biotin) is also required to maximize the yields of astaxanthin production. A culture medium containing partially-saccharified mussel-processing waste without supplementation (PSM) was also tested to study whether the salts and vitamins enhanced astaxanthin production. The composition of MS, PSM and PSMS is shown in Table 1.

*X. dendrorhous* was able to grow in all culture media, including the non-saccharified MS broth. The ability to use glycogen as a carbon source is a remarkable feature of the yeast that expands the information available about the ability of *X. dendrorhous* to metabolize polysaccharides. As mentioned above, the yeast ability to hydrolyze starch has been widely reported [23, 24]. However, to the best of our knowledge, this is the first report of glycogen hydrolysis by this microorganism.

**Fig. 3:**
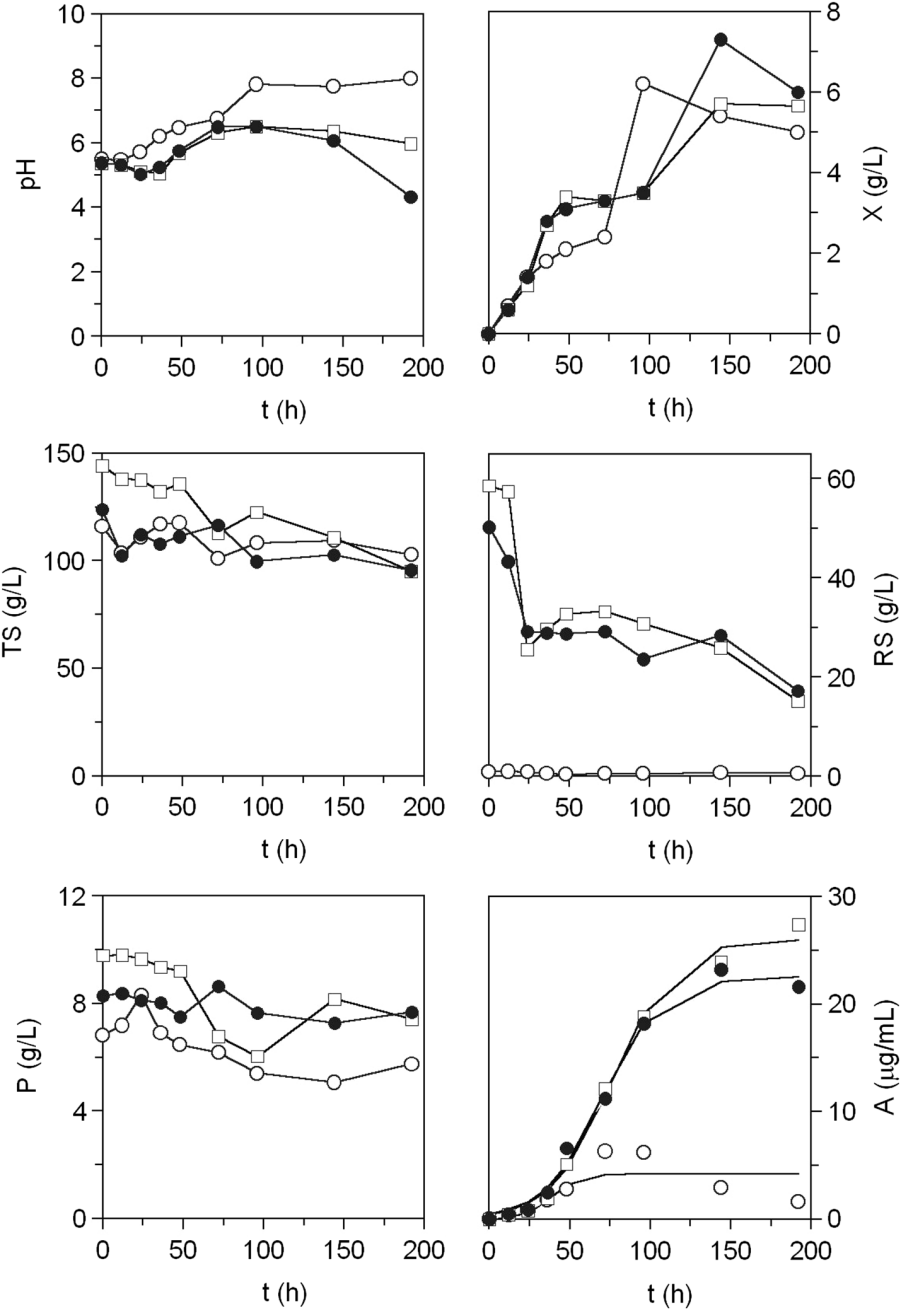
Cultivations of *X. dendrorhous* ATCC 74219 in non-saccharified (MS) and partially-saccharified mussel waste (PSM and PSMS). *Open circle* MS, *open square* PSM, *filled circle* PSMS. *X* biomass, *TS* total sugars, *A* astaxanthin, *RS* reducing sugars, *P* lowry-protein. Experimental data of astaxanthin are fitted to Eq. 2 (*continuous line*)

**Fig. 4:**
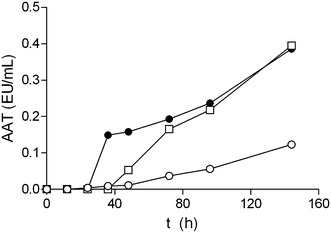
Amylolytic activity (EU/mL) of *X. dendrorhous* ATCC 74219 cultures grown in non-saccharified (MS) and partially-saccharified mussel waste (PSM and PSMS). *Open circle* MS, *open square* PSM, *filled circle* PSMS

Typical diauxic growth curves showing an initial phase of rapid growth followed by a lag phase after 36 h of culture, and reaching similar final biomass productions were observed (Fig. 3). Diauxic growth profiles evidenced a different pattern of sugar consumption throughout *X. dendrorhous* cultivation (Fig. 3). Mussel processing waste was partially hydrolysed with α-amylase, an enzyme exhibiting endo-acting activity that cleaves 1,4-d-glucosidic linkages between adjacent glucose units in long chain polysaccharides, like starch and glycogen, and yielding maltose as a final product. Furthermore, HPLC analysis of the culture media revealed very low initial glucose concentrations (3–4 g/L) in PSM and PSMS, and no glucose in MS medium. The low glucose content confirms expected α-amylase activity in a rich glycogen substrate and suggests most of initial reducing sugars, up to 50 g/L in partially-saccharified media (Fig. 1), are likely to be maltose and maltooligosaccharides. Diauxic growth was then due to a rapid consumption of di- and oligosaccharides hydrolyzed by *X. dendrorhous* extracellular α-glucosidase [24], according to the slight increase in sugar concentration observed (Fig. 3). Then a lag phase (36–96 h) in sugar consumption followed by a steep uptake during the last 48 h of culture (144–196 h) occurred. During this lag phase, yeast β-amylase was most likely induced by maltose [23], according with the observed increase in the extracellular amylolytic activity, which reached the highest activity levels (0.4 EU/mL) in the final stationary phase (Fig. 4). Amylolytic activity in glycogen-containing culture broth (MS) was lower and slower induced (Fig. 4). Low glycogen hydrolysis lead to lower sugar consumption compared to PSM and PSMS, and to higher yields on biomass produced in non-saccharified medium (Table 3). So, it is reasonable to assume cell biosynthesis in MS was due to protein intake (Fig. 3) that is also a carbon source, and to the highest phosphorus consumption observed in this medium (data not shown).

**Table 3:** Fitting parameters of astaxanthin production to Eq. (2) in non-saccharified (MS) and partially-saccharified mussel waste (PSM and PSMS)

Media	*A*_*m*_ (mg/L)	*v*_*m*_ (mg/L h)	*λ*_*a*_ (h)	*Y*_*A/X*_ (mg/g)	*Y*_*A/TS*_ (mg/g)	*Y*_*X/TS*_ (g/g)
MS	4.18 (NS)	0.126 (NS)	21.77 (NS)	0.320	0.122	0.381
PSM	26.00 ± 1.98	0.345 ± 0.082	38.98 ± 9.09	2.256	0.558	0.115
PSMS	22.50 ± 1.79	0.313 ± 0.078	34.15 ± 9.26	3.565	0.653	0.215

Yields of *X. dendrorhous* ATCC 74219 cultivation in MS, PSM and PSMS are also shown

*Y*_*A/X*_ yield of astaxanthin production on biomass, *Y*_*A/TS*_ yield of astaxanthin production on sugars consumed, *Y*_*X/TS*_ yield of biomass on sugars consumed, *NS* non significant

Astaxanthin productions were 5–6 times higher in saccharified than in non-saccharified media (Fig. 3; Table 3), reaching 26.0 in PSM and 22.5 mg/L in PSMS, respectively (Table 3). The improved astaxanthin production in mussel medium confirms a high C/N ratio promotes astaxanthin production, since this ratio was 35 in by-product formulated media and 6.3 in YPD (Table 1). Optimal productions in mussel by-product formulated media were higher than in Yucca broth containing date juice (60 % fructose and 40 % glucose) and urea as carbon and nitrogen sources (8.1 mg/L) [32], and in wood acid hydrolysate rich in xylose (10.4 mg/L) [18]. Culture media yielding similar productions were corn fiber hydrolysate containing glucose, xylose, arabinose and galactose as carbon sources (20 mg/L) and corn-starch hydrolysate [16], and corn steep liquor as carbon and nitrogen sources (25.3 mg/L) [33]. Supplementation of partially-saccharified mussel medium with salts and vitamins was found to increase the yields of astaxanthin production (Table 3) compared to non-supplemented medium (PSM).

These results then proved the suitability of mussel processing waste as a source for *X. dendrorhous* growth, although it needs saccharification for its use as a source of astaxanthin.

### Effects of the initial concentration of sugars on astaxanthin production

High concentrations of glucose have been reported to reduce astaxanthin production rate [34]. An experiment followed where the effluent was saccharified with α-amylase and glucoamylase. The joint action of these two enzymes has a synergic effect on the hydrolysis of glycogen, yielding glucose as the final product [19, 20]. The conditions utilized in the present research provided indeed a high degree of hydrolysis (92 %), leading to an effluent containing 129.5 g/L total sugars, 106 g/L reducing sugars, and 11 g/L protein.

Four nutritive broths were prepared by diluting the α-amylase and glucoamylase saccharified effluent to provide initial glucose concentrations of 20, 40, 80 and 100 g/L (Table 1). Totally-saccharified media were added with salts and vitamins because in addition to improving the yields of astaxanthin production, media supplementation was found to inhibit the slight cell lysis observed in non-supplemented medium (PSM).

**Fig. 5:**
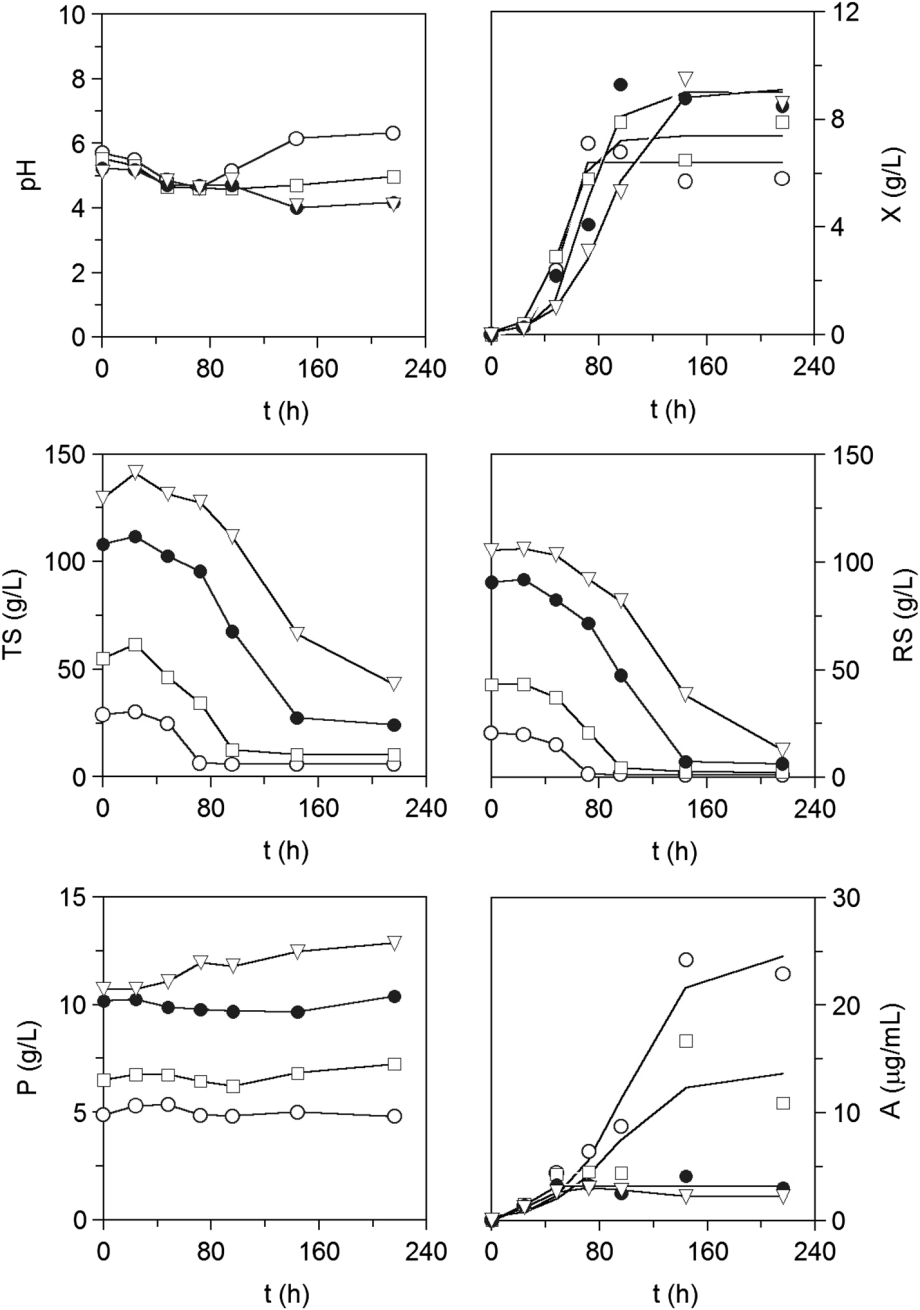
Cultivation of *X. dendrorhous* ATCC 74219 in totally-saccharified mussel waste (TSMS) containing different initial concentration of sugars. *Open circle* 20 g/L, *open square* 40 g/L, *filled circle* 80 g/L, *inverted triangle* 100 g/L. *X* biomass, *TS* total sugars, *A* astaxanthin, *RS* reducing sugars, *P* lowry-protein. Experimental data of astaxanthin and biomass are fitted to Eq. 2 (*continuous line*)

Compared to partially-saccharified media, no diauxic growth profiles were observed (Fig. 5). Maximal cell concentrations increased and maximum growth rates decreased with increasing glucose initial concentration (Table 4), following previously published results [34]. On the other hand, maximal astaxanthin productions were tenfold lower in culture medium containing 100 g/L glucose compared to 20 g/L initial sugar content (Table 4). Those differences are in agreement with the marked decrease in the yields of astaxanthin production to biomass with increasing initial sugar content (Table 4). These results confirm previous findings where *X. dendrorhous* undergoes Crabtree effect and other inhibitory effects derived from the high concentrations of glucose, and so high levels of oxygen are necessary to maintain high astaxanthin productivity [34]. The Crabtree effect consisted of glucose degradation proceeding partially via aerobic fermentation at high glucose concentrations and was previously reported for *P. rhodozyma* [28].

**Table 4:** Fitting parameters of biomass and astaxanthin production to Eq. (2) in totally-saccharified mussel waste (TSMS) containing different initial concentration of sugars

	Biomass			
	*X*_*m*_ (g/L)	*v*_*m*_ (g/L h)	*λ*_*X*_ (h)	*Y*_*A/X*_ (mg/g)
20 g/L	6.35 ± 1.03	0.977 (NS)	45.61 (NS)	3.962
40 g/L	7.41 ± 1.08	0.159 ± 0.119	30.78 ± 19.05	1.389
80 g/L	9.03 ± 1.74	0.185 ± 0.168	45.12 ± 24.91	0.355
100 g/L	9.14 ± 1.16	0.126 ± 0.060	50.77 ± 17.46	0.261

	Astaxanthin			
	*A*_*m*_ (mg/L)	*v*_*m*_ (mg/L h)	*λ*_*a*_ (h)	*Y*_*A/TS*_ (mg/g)
20 g/L	24.64 ± 6.53	0.272 ± 0.208	54.58 ± 32.25	1.001
40 g/L	13.71 ± 8.72	0.142 (NS)	42.96 (NS)	0.268
80 g/L	3.24 ± 0.80	0.419 (NS)	20.77 (NS)	0.036
100 g/L	2.58 ± 0.47	0.308 (NS)	20.18 (NS)	0.024

Also yields of astaxanthin on biomass produced (*Y*_*A/X*_) and on total sugars consumed (*Y*_*A/TS*_) are shown

*NS* non significant

## Methods

### Microorganisms

We used two wild-type *X. dendrorhous* strains CECT1690 and CECT11028 from the Spanish Type Culture Collection, and a recombinant strain from the American Type Culture Collection, ATCC 74219. Stock cultures were frozen stocks at −80 °C in Yeast Peptone Dextrose (YPD) broth containing 25 % (v/v) glycerol.

### Culture media

The screening of astaxanthin producer strains was carried out using two synthetic media, yeast peptone dextrose (YPD) and yeast complete medium (YCM). Astaxanthin production by *X. dendrorhous* was tested in four culture broths formulated using mussel-processing wastewater (MPW, Table 1). Proteins were removed by precipitation after acidification of MPW to a pH 4.0–4.5 and then followed by MPW concentration using 100 kDa ultrafiltration as previously described in detail [31]. The initial composition of concentrated MPW (CMPW) was: 4.22 ± 0.03 g/L of Lowry protein, 137 ± 7.69 g/L of total sugars and 0.69 ± <0.01 g/L reducing sugars. CMPW was partially-saccharified with α-amylase from *Aspergillus oryzae* (Sigma-Aldrich, St. Louis, MO). Total saccharification was performed by sequential addition of glucoamylase from *Aspergillus niger* (AMG 300L; Novozyme Nordisk, Bagsvaerd, Denmark) to the partially-saccharified reaction mixture [19]. Hydrolysis were carried out at a controlled temperature (45 °C) in a thermostatized bath under orbital agitation (100 rpm) for 18 and 24 h, respectively. The enzyme/substrate ratio was 3.5 U/mL for α-amylase and 0.3 U/mL for glucoamylase. The progress of the enzymatic reaction was assessed according to the degree of hydrolysis (H), expressed in percentage, defined as the ratio of reducing sugars (RS) in the supernatant and total sugars (TS) in the concentrated mussel effluent. According to this definition, 100 % hydrolysis corresponds with total conversion of glycogen to glucose [19]. Following hydrolysis, samples were centrifuged (12,000*g*, 15 min) and the supernatants recovered for analytical determinations and culture broth preparation. Table 1 shows the composition of the culture media. Yeast extract was from Cultimed (Panreac Química, Spain), and all salts and vitamins were analytical grade and purchased from Sigma-Aldrich (St. Louis, MO). All culture media were sterilized at 105 °C for 30 min.

### Culture conditions

Cell suspensions in sterile distilled water were adjusted, after a previous calibration between 700 nm OD measurement and direct haemocytometric counting, so that the initial populations of the cultures were 2 × 10^6^ cell/mL in each flask [31].

Submerged fermentations were carried out in 250 mL Erlenmeyer flasks containing 50 mL of production medium, being each flask an experimental unit for a defined incubation time. The experiments were carried out at 22 °C, 300 rpm orbital agitation and illumination with Sylvania F30W/GRO lamps (Allied Electronics, Inc., USA) for 6–9 days.

### Sampling and analytical methods

At pre-determined times, each experimental unit was centrifuged (3500*g*, 20 min). Total sugars, reducing sugars and protein were measured in the supernatant. The pellet was washed twice, resuspended in distilled water and divided into two aliquots. One aliquot was used for quantifying biomass by appropriate diluting and measuring the OD at 700 nm. After washing, biomass was dried at 105 °C and the dry weight calculated using a calibration curve. The other aliquot was used for astaxanthin extraction as described below. Total sugars (TS) were measured according to the phenol–sulfuric method of Dubois et al. [35], reducing sugars (RS) were determined by the 3,5-dinitrosalicylic acid (DNS) reaction [36] using glucose as standard, and protein using the method of Lowry [37]. Amylolytic activity (AAT) was measured using 4 % (w/v) starch solutions as the substrate, and expressed in enzymatic units (EU) as described by Murado et al. [31]. All analytical determinations were made in duplicate.

#### Cell disruption methods

Chemical, mechanical and enzymatic cell disruption methods based on previously published works [29, 30] were applied to the extraction of astaxanthin from *X. dendrorhous* grown in YPD for 72 h. Biomass (4 mL) was centrifuged (12,000*g*, 15 min) and cells disrupted using different pre-treatments described below in detail. Cell disruption assays were carried out in duplicate from biomass before and after drying in a desiccator under vacuum at ambient temperature for 24 h.

#### Chemical cell disruption with dimethyl sulfoxide (DMSO)

Biomass was treated with 1 (DMSO) or 2 mL (2×DMSO) preheated DMSO (55 °C) and vortex agitated for 30 s. Samples were sonicated (4 cycles of 5 min; P Selecta Ultrasons, Selecta S.A., Barcelona, Spain) avoiding overheating and pigment damage by cooling the sample between cycles in an ice-water bath.

#### Chemical cell disruption under acidic conditions

*X. dendrorhous* cells were resuspended in 4 mL 0.5 M HCl and autoclaved at 121 °C for 5 min. Samples were cooled to ambient temperature prior to astaxantin extraction.

#### Mechanical cell disruption using glass beads

Biomass was weighed and suspended in 2 mL 0.1 M phosphate buffer pH 7.0. Identical weight of acid-washed glass beads 425–600 µm (30-40 U.S. sieve; Sigma-Aldrich, St. Louis, MO) was added and vortex (Reax Top Vortex Mixer, Heidolph, Instruments GmbH & Co., Germany) mixed for 10 min.

#### Enzymatic cell disruption

Cells were resuspended in 2 mL 0.1 M phosphate buffer pH 7.0 and alcalase 2.4 L (Novozyme Nordisk, Bagsvaerd, Denmark) was added at a ratio of 0.01:1 (U/mL) enzyme/substrate. A solution of 10 % SDS was added, and proteolysis was carried out in a water bath with soft agitation at 40 °C for 60 min.

### Astaxanthin extraction

Astaxanthin was extracted by adding 2 mL of a mixture hexane:ethylacetate (1:1) and 0.01 M phosphate buffer pH 7.0, agitated for 30 s and separated by centrifugation (3500*g*, 5 min). The pigmented organic phase from the supernatant was recovered and the astaxanthin measured spectrophotometrically (PerkinElmer^®^ Lambda 25 UV/Vis spectrophotometer, PerkinElmer Inc., Massachusetts, USA) at the λmax (480 nm: A480). The spectrophotometric measurement at this wavelength is due to astaxanthin and other carotenoids. Nonetheless, *X. dendrorhous* contains astaxanthin as its main (>85%) pigment [38], being over-produced in the genetically-modified strain ATCC 74219 [27]. Therefore, A480 here reported is mainly due to astaxanthin.  Extractions were repeated twice, and pigment recovery calculated as follows [30]:

1 $$Y = \frac{{A_{480} \times V_{Hx:EtAc} \times 10^{6} }}{{100 \times V_{c} \times E_{1\% } }}$$

where *Y* is the astaxanthin per volume of culture (mg/L); *V*_*Hx:EtAc*_ is the volume of extracting solvent; *V*_*c*_ the volume of culture and *E*_1 *%*_ the specific extinction coefficient.

### Mathematical models

A logistic equation fitted the kinetics of biomass and astaxanthin production by *X. dendrorhous* [39]:

2 $$P = \frac{{P_{m} }}{{1 + \exp \left[ {2 + \frac{{4v_{m} }}{{P_{m} }}(\lambda_{p} - t)} \right]}}$$

where *P* is the biomass or astaxanthin production (mg/L); *t* is the time of culture (h); *P*_*m*_ is the maximum biomass or astaxanthin production (mg/L); *v*_*m*_ is the maximum rate of biomass or astaxanthin production (mg/L h), and *λ*_*P*_ is the delay in biomass or astaxanthin production (h).

### Numerical methods

Fitting procedures and parametric estimations were carried out by minimizing the sum of quadratic differences between observed and model-predicted values using the nonlinear least-squares (quasi-Newton) method provided by the Solver macro of the Microsoft Excel 2007 spreadsheet (Microsoft, Redmond, WA).

The yields of astaxanthin production on biomass (*Y*_*A/X*_) and sugars consumed (*Y*_*A/TS*_) were calculated by dividing the maximum values of astaxanthin production (mg/L) by the biomass (g/L) and total sugar consumption (g/L), respectively. Also, the yield of biomass on sugars consumed (*Y*_*X/TS*_) was calculated to compare the performance of the three strains of *X. dendrorhous* in YPD and YCM.

## Abbreviations

AAT: amylolytic activity
CMPW: concentrated mussel processing wastewater
DMSO: dimethylsulfoxide
EU: enzymatic units
H: degree of hydrolysis
MPW: mussel processing wastewater
MS: mussel-processing waste medium supplemented with salts and vitamins
PSMS: partially-saccharified mussel-processing waste medium supplemented with salts and vitamins
RS: reducing sugars
TS: total sugars
YCM: yeast complex medium
YPD: yeast potato dextrose
